# Targeted dream incubation at sleep onset can influence later dream content in REM sleep: a pilot study

**DOI:** 10.3389/frsle.2026.1812535

**Published:** 2026-06-24

**Authors:** Adam Haar Horowitz, Karen R. Konkoly, Michelle Carr, Robert Stickgold, Pattie Maes

**Affiliations:** 1 DUST Systems, San Francisco, CA, United States; 2 MIT Media Lab, Massachusetts Institute of Technology, Cambridge, MA, United States; 3 Department of Psychiatry, Center for Sleep and Cognition, Beth Israel Deaconess Medical Center, Boston, MA, United States; 4 Department of Psychology, University of Cambridge, Cambridge, United Kingdom; 5 Department of Psychiatry and Addictology, University of Montreal, Montreal, QC, Canada; 6 Center for Advanced Research in Sleep Medicine, CIUSSS-NIM, Montreal, QC, Canada; 7 Department of Psychiatry, Harvard Medical School, Boston, MA, United States

**Keywords:** dream, dream engineering, EEG, hypnagogia, rem, sleep

## Abstract

Targeted dream incubation (TDI) is a highly effective method for eliciting hypnagogic dreams related to specific topics through the presentation of verbal prompts and serial awakenings at sleep onset. In this pilot study, we tested whether TDI at sleep onset can effectively direct dream content in subsequent rapid eye movement (REM) sleep. We allowed participants a daytime nap opportunity following TDI at sleep onset. Serial awakenings were performed both at sleep onset and after entry into REM sleep. Our primary objective was to assess whether the TDI protocol during the sleep onset period would continue to affect dream content in REM sleep, producing dreams of the target content (“tree”) in the first REM awakening. Our second objective was to assess incorporation when participants received additional TDI prompts following REM awakenings. All 11 participants successfully incubated the target theme at sleep onset, and eight subsequently obtained REM sleep. Four of these participants (50%) incorporated the target theme into their first REM dream, and five incorporated the target theme in subsequent REM dreams (63%). Results provide preliminary evidence that TDI may impact dreams in REM sleep. This method of engineering dreams across sleep stages may be useful for understanding how dream generation and function may be continuous or different across sleep stages.

## 1 Introduction

Traditions across history and cultures show that dream content can be shaped by focusing on a topic before sleep with the intention to dream about it—a practice known as dream incubation (Patton, 2004; Mallett et al., 2024). Ancient Greeks, for example, slept in temples dedicated to Asclepius, the god of healing, hoping to receive therapeutic visitations from the deity in dreams (Patton, 2004). A Chester Beatty papyrus from Upper Egypt (c.1350 BCE) describes drawing Bes on the hand, wrapping it in consecrated cloth, and sleeping in silence to invoke the god's wisdom in dreams (Rees, 1966).

Experimental research offers a mixed view on the reliability with which incubation can influence dreams. On one hand, laboratory studies strongly indicate that presleep experiences influence dreams (e.g., Stickgold, 2001; Wamsley et al., 2010; Wamsley and Stickgold, 2019). The sleeping environment, an important feature of many ancient dream incubation practices, is also known to influence what one dreams about (Picard-Deland et al., 2021b; Horowitz et al., 2025). On the other hand, more research is needed to determine the extent to which intentional incubation before sleep can steer the specific topics of dreams later in the night. In two studies, participants were asked to incubate a dream about a current concern by silently repeating a question to themselves as they fell asleep, such as “Will I succeed on my exams?” (Saredi et al., 1997; Bisson and Baylor, 1994). Dreams pertaining to the target content were reported in both studies, but not more often than dreams pertaining to nonincubated concerns (Bisson and Baylor, 1994) or occurring on nights without incubation (Saredi et al., 1997). Other studies have found high rates of incorporation (>70% of participants) when a topic of personal interest was incubated for multiple consecutive days (Barrett, 1993; Cartwright, 1974). However, these studies did not compare incubation rates to control conditions, precluding conclusions about the extent to which on-topic dreaming resulted from incubation itself vs. other factors such as the continuity between waking and dreaming experiences (Schredl and Hofmann, 2003). Indeed, in a study in which participants were instructed to either incubate on their own current concern or the concern of a fellow participant, judges found no better than chance incorporation of the target material into overnight dreams (Griffin and Foulkes, 1977).

There have also been several studies showing that presleep suggestions administered by an experimenter can produce dream incorporation of target themes selected by experimenters (e.g., Barber and Calverley, 1962; Stoyva, 1961; Tart and Dick, 1970; Tart, 1964; Dement, 1974). While these studies did not refer to their procedures as incubation, the procedures did involve having participants focus on a topic before sleep to cultivate the intention or expectation to dream about it. Many of these studies included conditions where suggestions were preceded by a hypnotic induction (e.g., Stoyva, 1961; Barber et al., 1973; Barber and Calverley, 1962; Tart, 1964; Tart and Dick, 1970). Barber et al. (1973) found that suggestions worded directively (“You will dream of…”) were more effective when preceded by hypnotic induction, while permissive suggestions, more akin to current definitions of dream incubation (“Try to dream of…”), were more effective without a prior hypnotic induction.

While these prior studies found varying rates of incorporation across a night of sleep, the recently developed method of targeted dream incubation (TDI) reliably produces incorporation of the target content in sleep onset dreams in a single session (>90% of participants) (Horowitz et al., 2023; Youngren et al., 2025; Bellaiche et al., 2024). In this method, participants are repeatedly awoken to report their dreams during the transition between wake and sleep, a period also known as sleep onset, often corresponding to sleep stage N1. Upon returning to sleep, participants are reminded to think of the target topic, and they are reawakened between 1 and 5 min after the detection of sleep onset. This method likely yields reliable dream incubation because cognitive control is sufficiently preserved at sleep onset to allow for recall of task-relevant ideation (Horowitz et al., 2023). In addition, the method provides multiple opportunities to reactivate one's intention to dream of the target theme and emphasizes dreams reported in close temporal proximity to the reminder prompt. In an early account of how waking cognition shapes emerging dreams, Silberer (1951) reported that deliberate presleep cognition can shape dream content at sleep onset. A later study investigating the efficacy of incubation found that dream reports collected 10 min after sleep onset contained the highest levels of incorporation of the target theme compared to other sleep stages (Barber et al., 1973). Across converging evidence, Nielsen (2017) and Stickgold (2001) each demonstrate that waking thoughts, tasks, and intentions reliably intrude into hypnagogic imagery, showing that dream content can be deliberately and systematically incubated during the sleep-onset period.

Given the reliability of TDI for influencing sleep onset dreams, here we sought to conduct a preliminary investigation into the extent to which TDI may impact subsequent dreams in rapid eye movement (REM) sleep. Indeed, in one prior study investigating incubation across sleep stages, sleep onset incorporation predicted later on-topic dreams in the first period of rapid eye movement (REM) sleep (Barber et al., 1973). Further, dreams repeat similar themes throughout the night (Picard-Deland et al., 2023) so there is good reason to think that incorporation of a target topic at sleep onset might lead to later on-topic REM-sleep dreams. Past work from Cipolli et al. (2004) shows that incubation protocols resembling TDI, involving awakening from REM sleep and delivering presleep verbal cues before return to REM sleep, can successfully bias dream content even in REM sleep. This work leaves open the question of the influence of incubated hypnagogia on subsequent REM-sleep dreams. Given that our most immersive and memorable dreams tend to occur in REM sleep, this stage is an important target for developing effective techniques to influence dreaming. Further, understanding how dreams in hypnagogia affect later dreams throughout the night is important for understanding the functions of dreaming in different sleep stages. For example, Zadra and Stickgold (2021) hypothesize that sleep onset dreams help “tag” memories for later processing during REM-sleep dreams.

We performed TDI including several serial awakenings at sleep onset, and then provided a longer nap opportunity. Participants were then awoken for dream reports each time they entered REM sleep, and were reminded to think of the target theme each time they returned to sleep. Our findings suggest that TDI at sleep onset can produce incorporation of on-target themes into hypnagogic dreams, replicating prior findings (Horowitz et al., 2023; Youngren et al., 2025; Bellaiche et al., 2024), as well as into the REM-sleep dreams that occur subsequently. We also show evidence that incorporation of the target theme can be maintained with the combination of TDI at sleep onset and additional reminder prompts prior to re-entry into REM sleep. Our results provide preliminary evidence that TDI at sleep onset can impact REM-sleep dreaming, paving the way for future investigations of the interplay of engineered dream content across sleep stages.

## 2 Methods

We enrolled 16 healthy university students (seven female, nine male; Age = 20.56 ± 1.9 years) to participate in a daytime napping study. All participants were healthy adults with no diagnosed sleep disorders, no current use of sleep-affecting medications, and no known neurological or psychiatric conditions.

**Figure 1:**
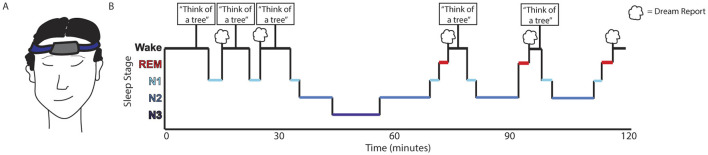
Experimental timeline. **(A)** First, participants arrived to the laboratory between 12 and 4 p.m. and were fitted with a Zmax Hypnodyne headband. **(B)** Illustrative example of a hypnogram. Each time participants were given an opportunity to fall asleep, they were prompted, “Remember to think of a tree.” During the first 30 min, participants were awoken 2–6 times from sleep onset to report their dreams. During the final 90 min of the nap, participants were awoken 2–3 times from REM sleep to provide dream reports.

Participants arrived at the laboratory in the afternoon between the hours of 12:00 p.m. and 4:00 p.m. Afternoon naps were chosen to optimize for the postprandial increase in sleepiness and to maintain consistency with prior TDI studies (Horowitz et al., 2020, 2023). Sleep restriction was not implemented prior to the nap due to participant burden and potential confounds related to elevated sleep pressure. Five participants were excluded from analyses for: (1) failure to fall asleep (*n* = 3); (2) failure to follow experimenter instruction (*n* = 1); or (3) bluetooth connectivity failure resulting in missing physiological data (*n* = 1). Participants were given a consent form to read and sign and were told the experiment investigated the relationship between rest and cognitive flexibility. After reading and signing consent forms, participants were fitted with a Hypnodyne ZMax headworn electroencephalegraphy (EEG) device manufactured by Hypnodyne Corp in Sofia, Bulgaria. Sleep opportunities totaled 120 min, and participants were awakened 2–6 times (mean = 2.28, SD = 1.25) during sleep onset and 0–3 times from REM sleep (mean = 1.91, SD = 1.3), depending on time constraints (Figure 1).

All participants began the experiment with TDI at sleep onset, using the Dormio system (Horowitz et al., 2020) to incubate the dream theme “Tree.” Upon lying down, the Dormio instructed participants to “think of a tree.” The system detected physiological changes associated with sleep onset using a combination of muscle tone, heartrate, and electrodermal activity (Horowitz et al., 2020). A variable timer instigated wakeups between 1 and 5 min after sleep onset, to allow participants to experience different depths of sleep. The Dormio system detected sleep onset using physiological proxies—muscle tone, heart rate, and electrodermal activity—rather than real-time EEG staging, and the dreams collected therein may have occurred in N1 or early N2 sleep. Participants in Dormio-detected sleep onset were awoken by a recorded verbal prompt informing them they were falling asleep (“You're falling asleep”). They were then asked to vocalize their experiences (“Please tell me, what's going through your mind”), and their dream report was recorded. Once participants finished speaking, the system asked about their sleep state (“And were you asleep?”), to which participants responded “Awake,” “Halfway,” or “Asleep.” The system then instructed them to think of the incubation target (“Remember to think of a tree”) and to return to sleep (“You can fall back asleep now”).

This loop of events was repeated for a total time of 30 min, enabling multiple transitions between wake and sleep onset, after which participants were given an additional 90-min sleep opportunity. When two contiguous epochs of REM sleep were scored according to visual inspection of the EEG, an experimenter triggered an awakening protocol, with prompts delivered through the computer speakers. Participants were asked to report the thoughts they were having (“Please tell me, what's going through your mind?”), and verbal responses were recorded. Once participants finished speaking, the system repeated the dream prompt (“Remember to think of a tree”), and participants were given the opportunity to return to sleep. Upon detection of each subsequent REM period (two contiguous epochs), the experimenter triggered another awakening following the same protocol. This process was repeated until the end of the nap opportunity. Participants were awakened by the experimenter at the end of the nap and completed an open-ended written report of all dream content they could remember across the entire session. After this, participants completed the alternative uses task (AUT), listing all the alternative uses they could think of for trees (results not reported here).

### 2.1 Sleep scoring

**Figure 2:**
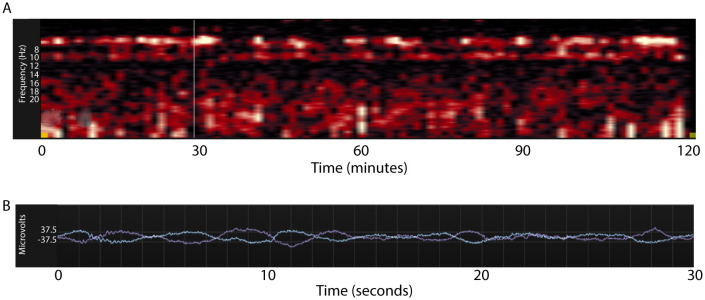
Representative sleep onset period from a study participant, as shown in the Hypnodyne ZMax software. **(A)** Power spectrogram (8–20 Hz) of frontal EEG throughout the entire 120-min nap. The vertical white line corresponds to the beginning of sleep onset, showing attenuation of alpha-band (8–12 Hz) activity. **(B)** EEG traces from F7 and F8 for the single 30-s epoch corresponding to the vertical white line in A. Note the emergence of slow rolling eye movements: conjugate, sinusoidal oscillations with initial deflection duration >500 ms, the canonical electrooculographic marker of sleep onset.

**Figure 3:**
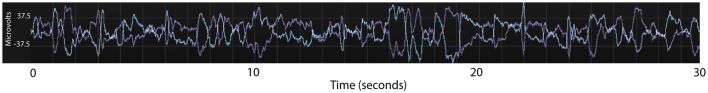
Representative REM-sleep epoch from a participant in this study. Frontal EEG and embedded ocular activity extracted from Hypnodyne ZMax channels F7 and F8 shows low-amplitude, mixed-frequency, desynchronized background activity with phasic bursts of rapid conjugate eye movements characteristic of REM sleep.

**Figure 4:**
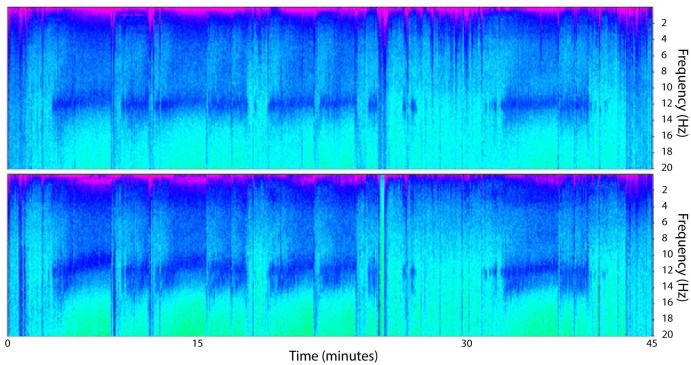
ZMax frontal-derivation signal fidelity against gold-standard polysomnography. **(Top)** power spectrogram derived from Hypnodyne ZMax channel F7 across a nap recording. **(Bottom)** power spectrogram derived from the F4 electrode of a simultaneously acquired standard polysomnography recording. The two spectrograms show concordant temporal evolution of EEG frequency content across the sleep cycle, including comparable delta- (0.5–4 Hz), theta- (4–8 Hz), and alpha-band (8–12 Hz) distributions.

Sleep was scored using a Hypnodyne ZMax EEG. Wireless transmission on the Hypnodyne uses a proprietary protocol to achieve a steady ultralow 3.5 ms latency. Hypnodyne streams two EEG channels, F7 and F8, both referenced to Fpz, with input impedances of 0.8–2.0 GΩ, a sampling rate of 256/s and a bandwidth of 0.1–128 Hz. Real-time visual sleep staging during experimental sessions was performed by experimenters trained in AASM scoring conventions, including completion of Hypnodyne's ZMax-specific scoring training program provided by the device manufacturer, which adapts staging criteria to the device's frontal montage. The Hypnodyne ZMax platform was developed for portable sleep research with REM-sleep detection as a primary design feature (Mota-Rolim et al., 2019). Rapid eye movements, low amplitude, mixed frequency EEG, the presence of increased 2–6 Hz activity in the form of sawtooth waves, and the absence of large bodily movements detected via Hypnodyne accelerometer were used as stage REM indicators. We note that this scoring approach deviates from standard AASM methodology, which requires central and occipital EEG derivations as well as EOG and EMG channels (American Academy of Sleep Medicine, 2023). During scorer training, manually identified REM periods were cross-validated against the device's retroactive automated REM-staging output to ensure concordance. Awakening decisions during nap sessions during the experiment itself were triggered by live visual inspection of streaming EEG by these trained scorers. Representative epochs scored as sleep onset and REM from participants in this study are provided in Figures 2 and 3, and Zmax spectrogram comparison to PSG is included in Figure 4 for reference.

### 2.2 Dream report scoring

Dream reports were scored by one rater. Dreams were considered to contain the target content if they contained an unambiguous mention of “tree” or part of a tree (including leaf, branch, root, or forest) (Horowitz et al., 2023). All instances of on-topic incorporation can be found in Table 1. Due to a technical error, the timing of awakenings was lost for the first four participants, so the elapsed time between subsequent reports reported here is the average of participants 5 through 11.

**Table 1:** All incorporations of the target theme reported during the study.

Participant	Sleep onset	First REM report	Subsequent REM
1	Report 3: A cobblestone pathway and the patterns of trees.	Report 4: The T shape of a tree.	
2	Report 4: Thinking about leaves and people.		
3	Report 2: In the woods.	Report 3: I'm in the tree.	
4	Report 1: Hanging out with my friend Payton. We picked Oranges from a tree. Report 2: The Ben Platt musical (tree boy).		Report 5: Going to an ice cream truck and climbing a tree with the friend who I went to the ice cream truck with.
5	Report 2: A teapot…also the tree because I remembered I had to think of the tree.		
6	Report 2: A friend and I playing tennis against a tree back and forth. But really it's this tree from this TV show I watched yesterday, and the two characters were buried underneath the tree.		
7	Report 2: Thinking about trees…I guess kind of like the like trees from game of thrones. Report 3: Thinking about like things I liked about trees in middle school.		
8	Report 1: Some graph theory scientist's building, this big complicated building made of upside down trees. I'm not quite sure what to do there…looks like a nice place for a party. Report 2: Binary trees. Search for real trees. A tree outside my apartment. People speaking to trees.	Report 3: Space tree like a spaceship but it's a tree.	Report 4: Moving around the tree. Laying on a giant leaf.
9	Report 1: Hanging out with Rejah. Hunt underneath a tree.	Report 3: Ice is about to kill somebody but then tree vines come.	Report 4: Gratitude…and big forests, taking back America.
10	Report 2: I'm playing the flute under a tree in my primary school.		Report 5: Someone is writing on a tree…and I am looking down at them doing it from my tall building…
11	Report 1: I have tingling in my chest and arms…and I feel like I'm floating. I had some thoughts of the tree…and then I completely forgot them. Report 2: I'm a fly and I can see through the fly eyes and I fly past the woman and almost get squished in the living room and then fly out to the tree in the backyard.		Report 4: I'm in a hammock among apple trees. Side to side. Report 5: I'm a giant and I threw a very large tree and it went through the atmosphere spinning and spinning and it became a rocket but still a tree with a mushroom top.

## 3 Results

### 3.1 N1 reports

**Figure 5:**
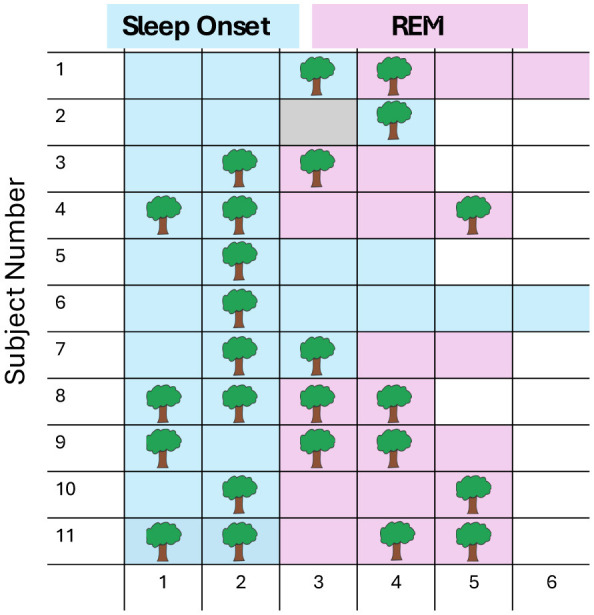
Shows incorporation of the target theme for each participant across reports collected following sleep onset (blue) and REM sleep (pink). Note that participant 2 had one awakening from N3 sleep due to experimenter error (gray shaded).

As shown in Figure 5 and Table 1, all 11 participants incorporated the theme “Tree” in sleep onset dream reports. On average, 57% of participants' dream reports collected from sleep onset contained the target theme (SD = 31%, range = 17%−100%, 15 of 31 total sleep onset reports). There was an average of 8.23 min between successive sleep onset reports (SD = 2.32, range = 6–12 min). The average intervals between successive sleep onset reports for participants 5–11 were 12, 9.6, 7, 10, 7, 6, and 6 min; timing data for participants 1–4 was lost due to a technical error, as noted above. The number of sleep onset awakenings per participant varied within the 30-min fixed window (range 2–6, mean 2.28), given the variability in how quickly participants transitioned to sleep, as well as the variable 1–5 min interval prior to awakenings. Examples of dreams that were not considered to incorporate the target theme include, “I think I saw pathways,” and, “There was something about a picnic and…writing on a whiteboard” and, “Typical material…carbon based. I can help with testing…and practicing tennis.”

### 3.2 REM reports

Eight of eleven participants (72%) obtained REM sleep and gave at least one REM dream report. Following their initial REM awakening, all eight of these participants then returned to REM sleep and were awakened 1–2 more times. The remaining three participants (27%) did not show polysomnographic signs of REM sleep in any part of the experimental period. As shown in Figure 5 and Table 1, in total, 7 of 8 participants who gave REM reports (87%) incorporated the target theme, and on average 50% of participants' dream reports collected following REM sleep contained the target theme (SD = 30%, range = 0%−100%, 10 of 21 total REM reports).

Of the eight participants who obtained REM dreams, 4 (50%) incorporated the target theme into their first REM dream. An average of 24 min elapsed between participants' last N1 report and their first REM report (SD = 3.21, range = 20–30 min, absolute values per participant are 25, 20, 23, 30, and 22 min). Five of these eight participants had incorporations into subsequent REM periods (63%). An average of 10.1 min elapsed between successive REM periods (SD = 3.97, range = 5–15, average time elapsed between REM periods per participant was 13, 15, 8, 5, and 9.5 min). Examples of dreams that were not considered to incorporate the target theme included, “Thinking about cooking somebody,” and, “My friend having wings but being afraid to fly and use them,” and, “Chemicals…dessert.”

## 4 Discussion

This pilot study replicates the high rates of successful dream incubation in sleep onset using TDI (Horowitz et al., 2023; Youngren et al., 2025; Bellaiche et al., 2024) and provides preliminary evidence that this method can seed the content of later REM-sleep dreams. Seven of eight participants who reached REM sleep incorporated the target theme in at least one REM-sleep report, and four did so in the first episode of REM sleep, indicating that targeted TDI at sleep onset may bias later dream content in REM sleep without additional prompting in many cases.

An exciting implication of this work is that it paves the way for studying how dreams at different points in the night interact with one another. It is known that dream themes recur throughout the night (Picard-Deland et al., 2023), but it is unknown the extent to which this is due to the continuity of dreaming with waking concerns or the causal impact of early dreams on later dreaming. Indeed, dream engineering methods often focus on manipulating concurrent dream mentation (e.g., Salvesen et al., 2024). Understanding the downstream effects of experimentally influenced dreams on later dreaming can help us understand whether dreams in different stages have interacting functions, such as whether sleep onset dreams “tag” memories for later processing (Zadra and Stickgold, 2021). A pattern worth noting in our sleep onset results is that more participants incorporated the target theme in their second sleep-onset period compared to their first (73% vs. 36%). This pattern is consistent with how TDI is hypothesized to operate: repeated wake-sleep transitions with reactivated target intentions progressively bias dream content toward the target theme. This cumulative pattern is consistent with prior TDI work (Horowitz et al., 2023). Future studies with larger samples could systematically test whether incorporation rates increase monotonically across successive sleep onset awakenings, and whether the effect of repeated incubation is cumulative across any stage of sleep.

This study also opens the door to using TDI for manipulating REM-sleep dreams. REM-sleep dreams can promote memory abstraction, procedural memory improvement, and emotional memory processing (Pereira et al., 2023; Picard-Deland et al., 2021a; Scarpelli et al., 2019). As such, they are an important treatment target, especially in nightmare disorder and PTSD. In the last two decades the literature on ways to manipulate REM-sleep dreams has blossomed, such as with lucid dreaming and sensory stimulation (e.g., Mallett et al., 2024; Konkoly et al., 2021; Salvesen et al., 2024). In contrast, there has been much less attention on the role of presleep intention on manipulating the content of REM dreams, other than for inducing lucid dreams or Imagery Rehearsal Therapy. We hope that this pilot study can inspire a resurgence of interest in the utility of presleep dream incubation for modifying dreams throughout the night. This method is also promising for translations because it can be easily implemented outside of the sleep laboratory. For example, TDI can be administered effectively using timers, without any physiological monitoring (Bellaiche et al., 2024). As such, individuals could use this method at home to curate the content of their dreams, benefitting both personal goals and mental health. Of note, we did not screen participants for mental imagery deficits such as aphantasia, which affects about 2% of the population (Zeman et al., 2015). If present in our sample, such deficits would have resulted in an underestimation of the true effect due to impoverished dream content in individuals with aphantasia (Dawes et al., 2020) as well as a reduced ability to visualize trees before sleep.

It will be interesting for future studies to compare how incubated themes may appear differently across sleep stages. Here, we applied the stringent criterion of considering “incorporation” as direct references to a tree or part of a tree. Studies with larger samples could perform more fine-grained analyses to test whether incorporation differs in form across sleep stages. For instance, REM sleep promotes weak semantic associations (Stickgold et al., 1999), so one might predict that target themes are incorporated in more indirect or abstract ways compared to sleep onset incorporations. We note the preliminary observation that some dreams collected from sleep onset appear similar in length to those in REM sleep (see Table 1), though we did not conduct statistical analyses of these comparisons. Future studies should investigate whether some apparent differences in sleep onset and REM-sleep dreams are due to the fact that REM periods are typically much longer than sleep onset transitions; awakening participants after only 1 min of REM sleep may have revealed dreams more similar to those in sleep onset. Future studies could further examine how the differing phenomenal content of dreams in different sleep stages affects the outcomes of incubated content. For instance, prior studies found that successful incubation in sleep onset can increase creativity as well as dream self-efficacy, a person's belief in their ability to alter the content of their dreams (Horowitz et al., 2023; Youngren et al., 2025). Perhaps incorporating target themes into REM-sleep dreams could further strengthen these benefits of TDI, given the immersive and associative narrative nature of many REM-sleep dreams.

This pilot study is subject to several limitations. Several sleep-staging constraints warrant acknowledgment. We used the Hypnodyne ZMax frontal-derivation headband rather than full polysomnography; while the ZMax has been validated against gold-standard PSG (Esfahani et al., 2023; Popovic et al., 2014; Myllymaa et al., 2016) and was developed with REM detection as a primary design feature (Mota-Rolim et al., 2019), it does not include the full electrode array specified by AASM guidelines (American Academy of Sleep Medicine, 2023). Awakenings from REM sleep were triggered by live visual scoring by trained scorers—a long-standing convention in serial-awakening dream research (Cipolli et al., 2004; Picard-Deland et al., 2023) but not equivalent to gold-standard staging done offline. Further, the Dormio system used physiological proxies for sleep onset rather than real-time EEG staging, and triggered awakenings 1–5 min after sleep onset detection, so the sleep onset dreams reported here likely occurred in N1 as well as early N2. Per-participant epoch-level sleep architecture across the full nap was not available from the original recordings for retrospective analysis, but it would be interesting for future studies to assess how the frequency and quality of incorporation may vary at different stages of sleep onset (Hori et al., 1994). We recommend that subsequent replications of this paradigm employ full PSG with offline scoring and report per-participant sleep architecture, and readers should interpret our sleep stage classifications with these constraints in mind. Additionally, the verbal prompt “You are falling asleep” delivered prior to dream reports may have influenced participants' subsequent self-reports of their sleep state. In addition, by design, participants are inherently aware of the TDI manipulation, and thus dream reports may be subject to demand characteristics. Further, we did not have any control groups, so we could not compare incorporation rates of the target theme to a baseline rate. However, given that we experimentally selected the target theme rather than incubating participants' current concerns, it is likely that baseline incorporations of trees would be much lower than the degree of incorporation observed. Indeed, in a prior TDI study, only 1.4% of reports referenced trees in the no-incubation group, whereas 70% of reports referenced trees in the group incubating on trees (Horowitz et al., 2023). We also were not able to disentangle the effect of repeated prompts delivered after REM awakenings from the effect of the initial TDI protocol during sleep onset. As such, it is unclear the degree to which TDI is efficacious for incubating REM dream content when delivered only in close proximity to a REM period.

Despite these limitations, this study suggests that TDI may incubate dream content not only at sleep onset, but also in REM sleep. This positions TDI as a promising method for engineering dreams throughout the night.

## Data Availability

The raw data supporting the conclusions of this article will be made available by the authors, without undue reservation.
